# A biophysically detailed model of the primary auditory cortex explains physiological forward masking, co-tuning of excitation and inhibition and cortical signal amplification

**DOI:** 10.1186/1471-2202-12-S1-P66

**Published:** 2011-07-18

**Authors:** Johan P Larsson, Ernest Montbrió, Gustavo Deco

**Affiliations:** 1 Computational Neuroscience Group, Universitat Pompeu Fabra, 08018 Barcelona, Spain; 2 Institució Catalana de Recerca i Estudis Avançats, 08010 Barcelona, Spain

## 

For a long time, studies argued for inhibition as the main mechanism responsible for two-tone suppression (a.k.a. forward masking) seen in primary auditory cortex (A1) neurons [1,2]. However, both computational [3] and experimental [4] papers afford a significant role to thalamocortical (ThC) synaptic depression in shaping the temporal response properties of A1. Also, the duration of inhibitory currents in A1 has been an issue of contention [6,7]. Another study of forward masking [5] used noise click stimuli to show that while responses to the probe were not fully recovered even 512 ms after presentation of the masker, inhibitory currents evoked by the masker lasted at most 100 ms, coinciding in duration with the complete suppression of probe responses. The authors proposed that a longer-lasting mechanism such as ThC or intracortical (IC) synaptic depression could complement inhibition by accounting for the lingering effect seen. They also demonstrated that pentobarbital anesthesia significantly prolongs inhibition, thus calling into question results such as [1,2]. Here we present a biophysically detailed, tonotopically organized network model of A1, which employs Hodgkin and Huxley neurons with stochastic synaptic depression in ThC synapses. Our model accounts for forward masking seen with both single tones [1,2] and noise stimuli [5], while showing approximately balanced excitation and inhibition [7-9]. Inspired by [10], we propose a plausible IC connectivity for the layers III and IV of A1, which selectively amplifies the broad input from the thalamus to yield the sharp frequency tuning seen in many studies of A1. We conclude that a combination of IC currents and ThC synaptic depression is imperative for accounting for the wealth of data seen in the neurophysiological literature, such as the phenomena we study here.
